# Supplementary motor area is deactivated during mental rotation tasks with biomechanical constraints in fMRI

**DOI:** 10.3389/fnhum.2024.1455587

**Published:** 2024-10-10

**Authors:** Makoto Nomura, Michihiko Koeda, Yumiko Ikeda, Amane Tateno, Ryosuke Arakawa, Yoichiro Aoyagi

**Affiliations:** ^1^Department of Rehabilitation Medicine, Graduate School of Medicine, Nippon Medical School, Tokyo, Japan; ^2^Department of Neuropsychiatry, Graduate School of Medicine, Nippon Medical School, Tokyo, Japan; ^3^Department of Pharmacology, Graduate School of Medicine, Nippon Medical School, Tokyo, Japan

**Keywords:** biomechanical constraints, functional magnetic resonance imaging, mental rotation, motor imagery, supplementary motor area

## Abstract

**Introduction:**

Mental rotation (MR) tasks of body parts involve anatomically interconnected brain systems. The systems are implicated in sensorimotor information integration and activate cortical motor-related areas, corresponding to the execution of similar motor tasks. In this study, we aimed to investigate the effect of varying the angle in the hand MR task on cerebral activation of the motor-related areas.

**Methods:**

Twenty healthy right-handed participants were recruited. We investigated cerebral activation while each participant decided whether a hand-palm image, rotated by 0°, 90°, 180°, and 270°, was a right or left hand.

**Results and discussion:**

A significant negative correlation between the angle and brain activity was observed in the right and left supplementary motor area (SMA) and right posterior anterior cingulate gyrus. The SMA was inactivated with 180°- or 270°-rotated images in the regions of interest analysis. 180°- and 270°-rotated palms would be biomechanically difficult to position; thus, SMA deactivation may be closely associated with biomechanical constraints. This study provided novel findings regarding the neurophysiological mechanisms of motor imagery and may be useful in developing treatment plans using MR tasks during patient rehabilitation.

## 1 Introduction

Motor imagery is a mental process that includes the rehearsal or simulation of an action by an individual. It is associated with the specific activation of neural circuits involved in the early motor control stages (Decety and Ingvar, 1990). These circuits include the supplementary motor area (SMA), primary motor cortex (M1), inferior parietal cortex, basal ganglia, and cerebellum (Decety et al., 1994). Thus, motor imagery supports motor learning and performance and may have a potential role in the development of neurorehabilitation strategies. Some studies have demonstrated that motor imagery training contributes to the reorganization of the motor network in stroke patients and is associated with improvements in upper limb function (Wang et al., 2020; Wang et al., 2023).

Mental rotation (MR) is a spatial ability defined as rotating two- or three-dimensional objects in imagination (Shepard and Metzler, 1971; Wexler et al., 1998). During the MR task, the mental activity of generating and manipulating images is evaluated by displaying two- or three-dimensionally rotated figures. For body-part MR tasks, a rotated image of a body part is presented. The participants then decide if the rotated image is the right or the left side of body part by mentally rotating and converting the image. Thus, MR requires the participants to be able to rotate and convert their mental images. Motor imagery ability can be evaluated by measuring the reaction time and correct answer rate (Moseley, 2004). MR tasks involving body parts have been used as a method of motor imagery treatment. In a randomized controlled trial of patients with phantom limb pain and complex regional pain syndrome, Moseley reported that pain relief was achieved using body-part MR tasks (Moseley, 2006). As MR tasks are implicit and simple, they can be used when pain interferes with accurate motor imagery.

The posterior parietal cortex and regions extending down into the superior posterior occipital cortex are consistently activated during body-part MR tasks in functional magnetic resonance imaging (fMRI) and positron emission tomography studies (Zacks, 2008). Activation of the superior parietal cortex during MR tasks indicates that it plays an important role in visuospatial image transformations (Cohen et al., 1996). Furthermore, a parametric increase in activation can occur with an increasing angle of rotation in the bilateral superior and inferior parietal lobules and right medial frontal gyrus during MR tasks involving alphanumeric characters (Gogos et al., 2010). Activity in the precentral cortex (including the SMA and M1) has been reported during MR tasks (Zacks, 2008). In addition, Nomura et al. (2024) reported that the hand MR task increased the excitability of spinal nerve function, as the F-wave amplitude and the persistence of the abductor pollicis brevis muscle increased during the task (Nomura et al., 2024). Thus, MR tasks activate movement-related areas from the brain to the spinal cord. Particularly, the SMA is known to be responsive during motor imagery tasks (Roth et al., 1996; Naito et al., 2002; Hanakawa et al., 2008; Guillot et al., 2009; Mizuguchi et al., 2013) and projects to both M1 and the spinal cord, playing an important role in motor control and simulation. This activity may reflect the use of motor simulations during MR tasks. A previous study using transcranial magnetic stimulation (TMS) has reported that TMS of the SMA facilitates MR performance, demonstrating the causative relationship between the MR performance and the SMA activation (Cona et al., 2017).

Thus, body-part MR tasks include anatomically interconnected brain systems involved in sensorimotor information integration, activating motor-related cortical areas corresponding to the execution of similar motor tasks. Although many studies have reported brain activity generation in body-part MR tasks (Vingerhoets et al., 2002; de Lange et al., 2005; Zacks, 2008), few have reported the association between activation of motor-related areas and the body image angle. Pamplona et al. demonstrated that in a hand MR task, increasing the rotation angle of the hand image tended to result in SMA deactivation. However, this relationship was not confirmed by correlation analysis, and thus remains unclear (Pamplona et al., 2022). If this association is elucidated, it could provide novel findings regarding the neurophysiological mechanisms involved in motor imagery. Therefore, in this study, we evaluated the effect of body image angle on the activation of motor-related areas during MR tasks. Methods for gaining insights into the localization of cognitive functions in the brain include EEG, fMRI, fNIRS, and more recently, simultaneous recording techniques (Ebrahimzadeh et al., 2022). In this study, we used fMRI due to its superior spatial resolution.

## 2 Materials and methods

### 2.1 Participants

We recruited 20 healthy right-handed participants aged 20–39 years, all with 15 or more years of education to control for the effect of educational background on the MR tasks (Moè et al., 2021). Additionally, all participants were medical staff who had graduated from a university or vocational school, working at a university hospital. Right-handedness was determined using the laterality quotient based on the Edinburgh Handedness Inventory. We measured the motor imagery ability of the participants using the vividness of movement imagery and the kinesthetic and visual imagery questionnaires. This study was approved by the Central Ethics Committee of the Nippon Medical School (approval #M-2021-3). All the participants provided written informed consent to participate in the study. All the experiments were conducted in accordance with the principles of the Declaration of Helsinki.

### 2.2 Mental rotation task

**FIGURE 1:**
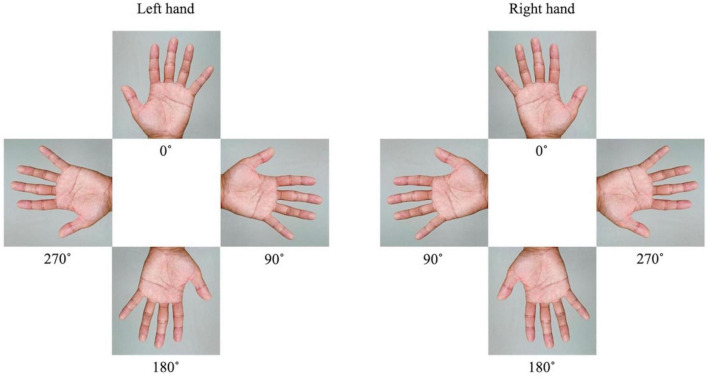
The images of a rotated palm and associated angles. The left and right images were rotated by 0°, 90°, 180°, and 270°.

**FIGURE 2:**
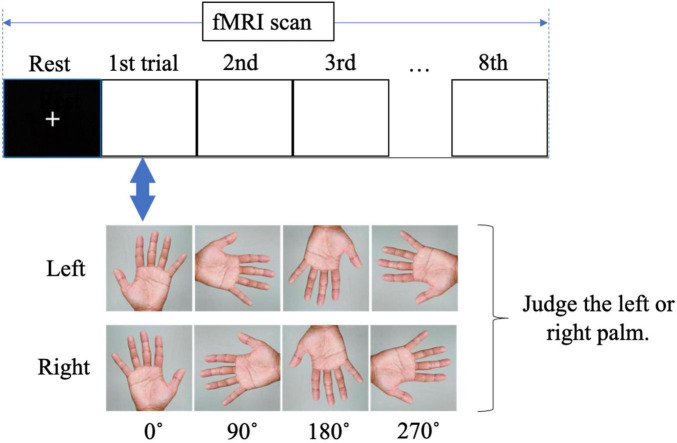
Visual of fMRI during the mental rotation task. The participants were asked to judge whether the palm image on the monitor was a right or left palm.

The MR task was performed in an MRI room by presenting an image of the hand on a monitor that could be viewed through a mirror while lying supine, and brain activity was imaged during the task. The MR task was created and presented on a personal computer using E-Prime 2.0 (Psychology Software Tools, USA). We used images of a rotated palm; when the middle finger was pointed upward, the image was said to be at 0°. The left and right images were rotated by 0°, 90°, 180°, and 270° (Figure 1). Each trial consisted of eight randomly presented images. The presentation time of the images was 5.9 s, and a fixation cross was randomly presented for 7.9, 8.4, or 8.9 s between the images. The trial was repeated eight times. The total fMRI time for the MR task was approximately 16 min. The participants were asked to identify whether the image on the monitor was a right or left palm. Once they had decided, the participant would press the corresponding button on either side of their body (Figure 2). After the task, reaction time and accuracy were recorded, and the average of these values was calculated. Accuracy was defined based on the participants ability to correctly select the right or left palm during the MR task. A higher accuracy value relates to an increased number of correct selections.

### 2.3 Functional MRI acquisition

Images were acquired using an Intera Achieva 1.5 T Nova scanner (Philips Electronics, The Netherlands). Functional images of 462 volumes were acquired using T2*-weighted gradient-echo planar imaging sequences sensitive to blood oxygenation level-dependent contrast. Each volume consisted of 31 transaxial contiguous slices with a 4-mm slice thickness + 1-mm gap, covering almost the entire brain (flip angle, 90°; time to echo [TE], 30 ms; repetition time [TR], 2000 ms; matrix scan, 64; field of view, 256). High-resolution T1-weighted anatomical images were acquired with the following parameters: flip angle, 8°; TE, 4.6 ms; TR, 9.3 ms; matrix scan, 256 × 256; field of view, 250 mm; and slice thickness = 1.2 mm, 160 slices.

### 2.4 Image analysis

The imaging data were preprocessed and analyzed using SPM12 (Wellcome Centre for Human Neuroimaging, UK) in MATLAB (MathWorks, USA). Structural T1 and functional images were manually reoriented to the anterior–posterior commissure line. To correct for between-scan movements, the functional images were realigned with the first image of each session. Functional images were spatially normalized to the standard space defined by the Montreal Neurological Institute (MNI) template. After normalization, all the scans were resampled to a resolution of 2 mm × 2 mm × 2 mm. The significance of hemodynamic changes was examined using a general linear model with boxcar functions convoluted with a hemodynamic response function at each image angle.

After fMRI preprocessing for each participant, group analysis (second-level analysis with SPM12) of the fMRI data was performed. The fMRI data were analyzed using the full factorial design as implemented in SPM12. A two-way repeated measures ANOVA model was conducted to examine the main effects and interactions of the laterality and the four angles in brain activity during the MR task. The statistical threshold was set as follows: *p* < 0.005, uncorrected; extent threshold, 20 voxels; and cluster-level correction for multiple comparisons at *p* < 0.05. We also performed a correlation analysis between the angle and brain activity. The statistical threshold was set as follows: *p* < 0.05; extent threshold, 100 voxels. Moreover, analysis of the regions of interest (ROI) was used to verify the difference between the results for 0° and other angles. Based on a previous study (Pamplona et al., 2022), an eight-mm-radius spherical ROI was set from the location (MNI coordinates, x, y, z) (16, −8, 58) with the most activation in the correlation analysis. Using the motor cortex template (Mayka et al., 2006), we identified this MNI coordinate as the right SMA. Activation in the ROI was evaluated for each condition using paired *t*-tests, with *p* < 0.005 uncorrected; extent threshold, 20 voxels; and cluster-level correction for multiple comparisons at *p* < 0.05.

### 2.5 Statistical analysis

A nonparametric method was used for statistical analysis of the reaction time and accuracy, as the normality of the obtained data was not confirmed. Therefore, a Friedman test was first conducted. Since significant differences were observed, a post-hoc Wilcoxon signed-rank test with Bonferroni correction was applied for each condition. The significance level was set at *p* < 0.05. SPSS Statistics 26.0 (IBM, USA) was used for all the analyses.

## 3 Results

### 3.1 Basic participant characteristics

Overall, 20 participants (10 men and 10 women; mean age, 26.8 ± 4.4 years) were initially recruited. All 20 participants met the inclusion criteria. The basic participant characteristics are presented in Table 1.

**TABLE 1:** Characteristics of participants.

	Mean (SD), *n* = 20
Age (years)	26.8 (4.4)
Gender (male: female)	10:10
EHI score	97.0 (6.6)
VMIQ score (1st person perspective)	33.0 (13.6)
VMIQ score (3rd person perspective)	31.7 (11.2)
KVIQ score (1st person perspective)	46.9 (6.9)
KVIQ score (3rd person perspective)	45.4 (9.4)

SD, standard deviation; EHI, Edinburgh Handedness Inventory; VMIQ, The Vividness of Movement Imagery Questionnaire; KVIQ, The Kinesthetic and Visual Imagery Questionnaire.

### 3.2 Reaction time and accuracy

**FIGURE 3:**
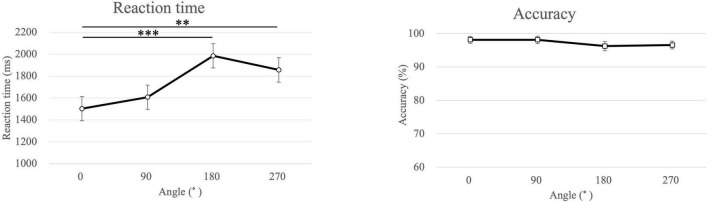
Mean of the reaction time and the accuracy for each angle in the mental rotation task. ***p < 0.001, **p = 0.002.

The average reaction times of the participants in the MR task were 1737 ± 573 ms for all angles of both hands, 1773 ± 589 ms for the left-hand images, and 1702 ± 564 ms for the right-hand images (Figure 3). The reaction time was significantly longer for the left-hand images than that for the right-hand images (*p* = 0.021). The reaction times were 1502 ± 482, 1607 ± 615, 1984 ± 711, and 1856 ± 694 ms for the 0°, 90°, 180°, and 270°-rotated images, respectively. Furthermore, the reaction time was significantly longer for the 180° and 270°-rotated images than that for the 0°-rotated images (180° > 0°: *p* < 0.001, 270° > 0°: *p* = 0.002).

The average accuracy in the MR task for all, left-handed, and right-handed images was 97.3 ± 3.4, 97.3 ± 3.8, and 97.2 ± 3.8 %, respectively. The average accuracy for 0°, 90°, 180°, and 270° was 98.1 ± 4.6, 98.1 ± 5.0, 96.3 ± 6.2, and 96.6 ± 5.2 %, respectively. No significant differences were observed in any of these comparisons.

### 3.3 The analysis of a two-way repeated measures ANOVA

Table 2 shows the main effect of each angle in a two-way repeated measures ANOVA. The main effect of each angle was observed in the right inferior frontal gyrus, the right superior temporal gyrus, the right parahippocampus, and the right anterior cingulate cortex (*p* < 0.05). Table 3 shows the main effect of the laterality in a two-way repeated measures ANOVA. The main effect of the laterality was observed in the right medial frontal gyrus, the right precentral gyrus, the left precentral gyrus, and the left posterior cingulate cortex (*p* < 0.05). Table 4 shows the interaction effect between laterality and angle in a two-way repeated measures ANOVA. The interaction between the laterality and angles was observed in the left medial frontal gyrus (*p* < 0.05).

**TABLE 2:** Main effect of angle in a two-way repeated measures ANOVA.

Brain regions	MNI coordinate	BA	F	*p*
R IFG	(44, 38, 12)	46	9.44	0.003
R STG	(26, 8, −26)	38	7.29	0.020
R parahippocampus	(10, −38, 2)	30	6.77	0.003
R ACC	(8, 42, −6)	32	5.99	0.004

MNI, Montreal Neurological Institute; BA, Brodmann area; R, right; IFG, inferior frontal gyrus; STG, superior temporal gyrus; ACC, anterior cingulate cortex.

**TABLE 3:** Main effect of laterality in a two-way repeated measures ANOVA.

Brain regions	MNI coordinate	BA	F	*p*
R MedFG	(10, −26, 54)	6	29.48	<0.001
R PrCG	(50, −8, 30)	6	22.41	<0.001
L PrCG	(−32, −28, 62)	4	17.68	<0.001
L PCC	(−24, −66, 8)	30	17.01	0.024

MNI, Montreal Neurological Institute; BA, Brodmann area; R, right; L, left; MedFG, medial frontal gyrus; PrCG, precentral gyrus; PCC, posterior cingulate cortex.

**TABLE 4:** Interaction effect between laterality and angle in a two-way repeated measures ANOVA.

Brain regions	MNI coordinate	BA	F	*p*
L MedFG	(−10, 66, 2)	10	29.48	<0.001

MNI, Montreal Neurological Institute; BA, Brodmann area; L, left; MedFG, medial frontal gyrus.

### 3.4 The correlation analysis between the angle and brain activity

**FIGURE 4:**
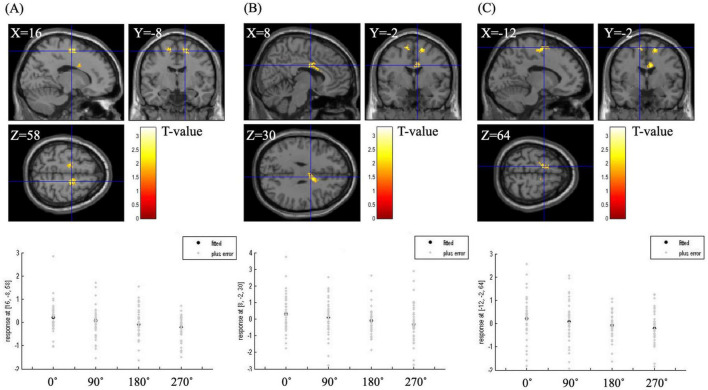
BOLD signal during the mental rotation task under the correlation analysis between angle and brain activity. The right supplementary motor area **(A)**, the right posterior anterior cingulate gyrus **(B)** and the left supplementary motor area **(C)** were significantly associated with angle (*p* < 0.05, extent threshold = 100 voxels). The graph below the figure is a plot diagram of each angle for each participant in the mental rotation task. As the angle increased, the activation of the three regions decreased.

Table 5 shows the brain regions during the MR task in the correlation analysis between angle and brain activity. A significant negative correlation was observed in the right and left SMA and the right posterior anterior cingulate gyrus (*p* < 0.05). The plot diagrams of each angle for each participant in the MR task are shown in the bottom lane in Figure 4. As the angle increased, the activation of the three regions decreased.

**TABLE 5:** Brain regions during the mental rotation task under the correlation analysis between angle and brain activity (*p* < 0.05, extent threshold = 100 voxels).

Brain regions	MNI coordinate	BA	T
R SMA	(16, −8, 58)	6	3.31
R posterior ACG	(8, −2, 30)	24	2.70
L SMA	(−12, −2, 64)	6	2.69

MNI, Montreal Neurological Institute; BA, Brodmann area; R, right; L, left; SMA, supplementary motor area; ACG, anterior cingulate gyrus.

### 3.5 The analysis of the ROI

**FIGURE 5:**
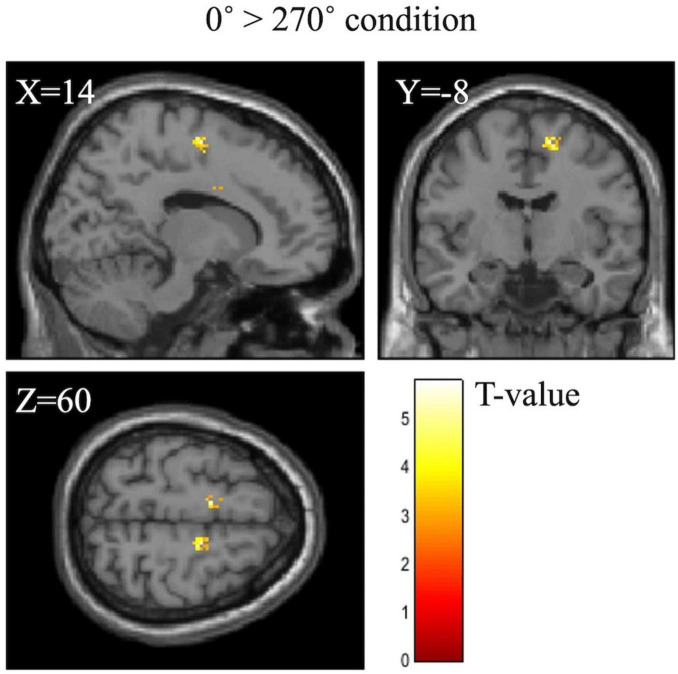
BOLD signal during the mental rotation task under the 0° > 270° condition. The 270° condition was associated with significantly less activation than the 0° condition in the right supplementary motor area.

Table 6 shows the activation of the right SMA at the difference between 0° and each angle. The right SMA was significantly activated under the 0° > 180° (*p* = 0.027) and 0° > 270° conditions (*p* = 0.001). Figure 5 shows cerebral activation during the MR task under the 0° > 180° or 270° condition. The 180° and 270° conditions were associated with significantly less cerebral activation than the 0° condition in the right SMA.

**TABLE 6:** Right supplementary motor area regions activated by each condition.

Condition	MNI coordinate	BA	T	*p* (FWE-corrected)
0°	(16, −10, 60)	6	5.72	0.002
90°	−	−	−	−
180°	−	−	−	−
270°	−	−	−	−
90° > 0°	−	−	−	−
180° > 0°	−	−	−	−
270° > 0°	−	−	−	−
0° > 90°	−	−	−	−
0° > 180°	(14, −8, 60)	6	3.85	0.044
0° > 270°	(14, −8, 60)	6	5.77	0.008

MNI, Montreal Neurological Institute; FWE, familywise error rate; BA, Brodmann area.

## 4 Discussion

This study investigated the effect of the angle of rotation on the activation of movement-related areas of the brain during hand MR tasks. We hypothesized that the activation of movement-related areas would increase as the rotation angle increased. However, significantly reduced activation of the SMA was observed when the participants viewed the 180° or 270° image compared with the 0° image, indicating that the movement-related areas were inactivated by specifically rotated hand-palm images.

### 4.1 Neural mechanism in MR tasks

Most studies suggest that the brain is activated in various regions during MR tasks, including the inferior parietal lobule, superior parietal lobule, precentral gyrus, inferior frontal gyrus, middle frontal gyrus, SMA, insula, inferior occipital gyrus, middle occipital gyrus, and cerebellum (Zacks, 2008). Particularly, hand MR tasks activate the bilateral superior parietal lobules, visual areas, and motor cortices, which are the brain regions related to motor imagery. Additionally, an interaction between the laterality and angles was observed in the left medial frontal gyrus in this study. A recent study has reported that this region is involved in the recognition of MR tasks (Tomasino and Gremese, 2015). To our knowledge, there have been no reports indicating that brain function differs when imagining the angles of left and right hands. The findings of this interaction in this study suggest that the left medial frontal gyrus is involved in recognizing finer differences in the angles and the laterality.

Furthermore, many studies have reported the activation of movement-related areas during MR tasks (Vingerhoets et al., 2002; de Lange et al., 2005; Zacks, 2008). When comparing brain activity during hand and letter MR tasks, de Lange et al. reported that the activation of motor-related regions was observed only during the hand MR task (de Lange et al., 2005). Among the movement-related areas, the SMA has been reported to coordinate movement through descending fibers that innervate the M1 to the spinal cord. Additionally, SMA is reportedly significantly involved in motor imagery (Hardwick et al., 2018; Fukumoto et al., 2021). A meta-analysis has reported SMA activation during body-part MR tasks (Zacks, 2008), with consensus on SMA activation. Moreover, Cona et al. demonstrated a causal relationship between the MR performance and SMA activation using TMS (Cona et al., 2017). Thus, the SMA may also have been activated by hand rotation imagery in this study, evoking motor hand imagery. However, the increased hand rotation angle was biomechanically difficult to assume, thus making visualization of the hand rotation complicated, potentially leading to the inactivation of motor-related areas.

### 4.2 Deactivation of SMA during MR tasks

Some studies have reported that MR increased brain activity in parietal regions depending on the orientation of the stimulus (Carpenter et al., 1999; Wilson and Farah, 2006). During MR tasks with alphanumeric characters, the superior parietal lobule, inferior parietal lobule, and medial frontal gyrus were reportedly activated with increasing rotation angles (Gogos et al., 2010). This suggests that MR with alphanumeric characters largely involves the parietal lobule involved in object identification and spatial cognition. Pamplona et al. investigated the correlation between three angles (0°, 90°, and 180°) and brain activity in hand MR tasks with haptic or visual stimuli (Pamplona et al., 2022). They reported a positive linear relationship in the left middle frontal gyrus with haptic stimuli and in the right superior occipital gyrus with visual stimuli. However, the analysis did not include the outward rotation of 270°, and no correlation with the motor area was observed. The novelty of this study is that it revealed a negative correlation between SMA activity and angle in MR tasks with palm images.

In this study, activation of the SMA decreased as the angle of the palm image increased. Additionally, the SMA was inactivated with 180°- or 270°-rotated images in the ROI analysis. Parsons reported that body-part MR tasks are affected by biomechanical constraints based on delayed reaction time within tasks that use pictures with limbs bent in unnatural directions (Parsons, 1987). Moreover, Guillot et al. measured brain activity during kinesthetic motor imagery (motor imagery from a first-person perspective) and visual-motor imagery (motor imagery from a third-person perspective) (Guillot et al., 2009). The study reported activation of motor-related areas and the inferior parietal lobule during kinesthetic motor imagery and activation of occipital lobe areas and the superior parietal lobule during visual-motor imagery. Furthermore, directly comparing the kinesthetic and visual-motor imagery revealed strong activation of the anterior and posterior SMA. Therefore, when the participants perceived the 180°- or 270°-rotated images in this study, we propose that the SMA may have been inactivated due to the biomechanical difficulty of attaining the limb position, meaning motor imagery from a first-person perspective was not recalled.

Previous studies revealed that young people perform hand-MR task for images of the back of the hand predominantly using recognition of the visual domain, and for palm images with recognition of the motor domain (ter Horst et al., 2010; Bläsing et al., 2013; Zapparoli et al., 2014). In this study, as we used palm images, it is likely that recognition of the motor domain was more strongly involved. Comparisons between visual and motor imagery have demonstrated that motor imagery is more likely to activate the SMA (Hamada et al., 2018). The findings of this study suggest that the decreased SMA activation could be due to a preference for visual processing over motor imagery. Furthermore, Qu et al. reported that congruent drawings of hands evoked significant activation in the SMA compared to incongruent drawings of hands, suggesting that biomechanical constraints affect the cognitive process of MR (Qu et al., 2018). The results of our study support these findings, indicating that the neural mechanisms of hand MR may vary depending on the angle of rotation. The results suggest that humans employ different strategies to efficiently perform MR tasks involving body parts.

### 4.3 Limitations

The rotation of the body images in this study was in a two-dimensional space that was easy to imagine, and the images were presented in a visually clear manner. The quality of visual input can modify the cognitive strategy used to solve hand MR tasks (Giovaola et al., 2022). Therefore, the simplicity of the task or the quality of visual input may have caused SMA deactivation. Further research is required to assess the brain activity changes with increasing task difficulty or with less visible stimuli. Additionally, the findings of this study are limited to right-handed young people aged 20–39 years and cannot be generalized to all age groups and patient populations. Additional studies are needed to characterize the changes in the activity of motor-related areas with MR tasks in left-handed individuals, ambidextrous individuals, and patients undertaking rehabilitation programs. Moreover, this study did not consider the influence of female sex hormone fluctuations on MR performance (Scheuringer and Pletzer, 2017; Bernal et al., 2020; Moè et al., 2021; Bernal and Paolieri, 2022; Gurvich et al., 2023). Future research should consider this factor to gain a more comprehensive understanding.

## 5 Conclusion

This study revealed that the SMA is deactivated during an MR task with biomechanical constraints. This study provided novel findings that elucidate the neurophysiological mechanisms involved in motor imagery. Additional evidence, including patient recruitment and demonstration that the key brain areas associated with MR tasks can serve as target areas for brain stimulation, is required to extend these findings to clinical settings.

## Data Availability

The raw data supporting the conclusions of this article will be made available by the authors, without undue reservation.
